# Encounters with adenovirus

**DOI:** 10.1080/03009734.2019.1613698

**Published:** 2019-05-29

**Authors:** Ulf Pettersson

**Affiliations:** Department of Immunology, Genetics and Pathology, Uppsala University, Uppsala, Sweden

**Keywords:** Adenovirus, proteomics, transcriptomics

## Abstract

In this paper I describe aspects of work on the human adenoviruses in which my laboratory has participated. It consists of two sections—one historic dealing with work performed in the previous century, and one dealing with the application of ‘omics’ technologies to understand how adenovirus-infected cells become reprogrammed to benefit virus multiplication.

## Looking backward

### Beginning a life in science

In 1965, when I was in in medical school, I contacted my pathology professor, Jan Pontén, asking if I could join his research group. Jan informed me that he was oversubscribed and instead recommended that I contact his colleague and friend, Lennart Philipson, who was working further down the hall in the old pathology building in Uppsala. Lennart had just returned from a postdoctoral training period with Igor Tamm at the Rockefeller. As luck would have it, he accepted me as a student and suggested that I work on the adenovirus (Ad) antigens, then known as the A-, B-, and C-antigens. Ads had been independently discovered by Rowe et al. (1) and by Hilleman et al. (2) about 10 years earlier. It was recognized from the start that the viruses replicated with exceptional efficiency and produced large amounts of soluble antigens. Philipson, who was closely allied with the Institute of Biochemistry in Uppsala (which under the leadership of Nobel laureate Arne Tiselius had become the Mecca of protein separation technology), was hoping to apply state-of-the-art biochemistry to virus studies. He predicted that Ads would be ideal for studies of virus components.

**Figure:**
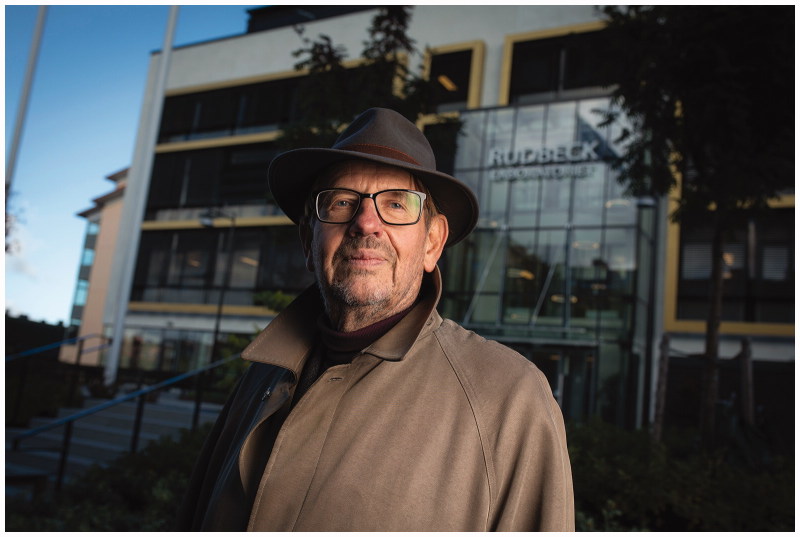
Professor Ulf Pettersson, winner of the Medical Faculty of Uppsala University Rudbeck Award 2018 ‘for his pioneering scientific achievements in virology and human genetics’.

**Figure 1.**
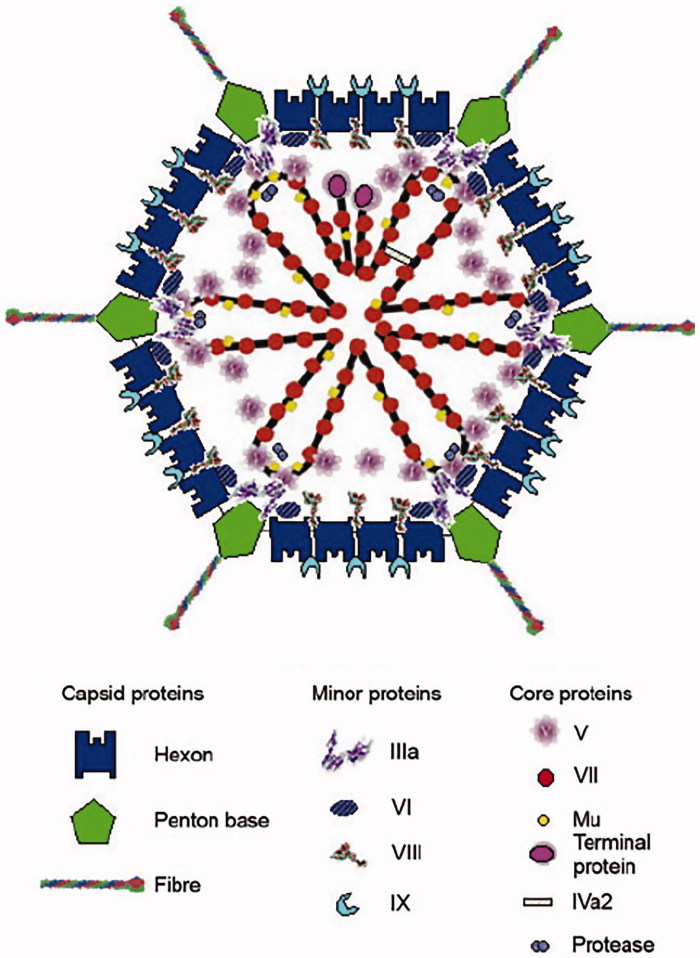
A schematic picture of the adenovirus particle showing the locations of the different capsomeres. Modified from (4).

At the time of my entry into research, Ad was a largely unknown entity. In fact, reprints of all published papers fitted into my briefcase. In 1965, however, a seminal paper appeared in the *Journal of Molecular Biology* authored by Valentine and Pereira (3). Using electron microscopy, they showed that the adenovirion has a strikingly handsome architecture. An icosahedral capsid, composed of 240 capsomeres, it carries antenna-like projections from each of the 12 corners (Figure 1). It became apparent from the structure that the three known soluble A-, B-, and C-antigens correspond to components in the viral shell. The A-antigen makes up the facets, and the B-antigen sits at the vertices. The B-antigen is composed of the vertex capsomere, which is non-covalently linked to an antenna. The last-mentioned is the C-antigen. Erling Norrby working with another serotype came to an identical conclusion and showed in addition that the B-antigens could form so-called dodecons consisting of 12 penton units (5). In 1966 Ginsberg et al. proposed a nomenclature for the antigens (6). Antigen A is named Hexon, whereas Penton is the name of the vertex capsomere consisting of the penton base with the non-covalently attached fiber, the C-antigen. The fiber and the penton base play key roles in the early virus–cell interactions. The knob of the fiber attaches the virus to its receptor. The receptor for most human Ad serotypes is CAR (coxsackie adenovirus receptor). An unexpected discovery was that two unrelated viruses use a common receptor reported before virology became molecular. Also the penton base plays an essential role in virus attachment through the presence of an RGD motif which interacts with integrins starting the reorganization of the cell structure.

Soon after its discovery it was recognized that the Ad family comprises several so-called serotypes and that these are associated with different symptoms in infected patients. Today more than 70 human Ads are known. In general, they cause mild disease, and subjects are often unaware of their encounters with the virus. The infectivity is low, and it was noted early that widespread infection often occurs in closed communities, e.g. among military recruits. The most common adenovirus infections are associated with mild respiratory symptoms. Other types cause keratoconjunctivitis or gastrointestinal and urinary tract infections. In rare cases Ads do cause life-threatening disease, primarily in immune-compromised patients. The Ad family is, based on antigenicity and DNA sequence homology, divided into seven subgenera, named A–G. Members of the different subgenera usually cause different symptoms. Trentin et al. conducted a systematic study of known human viruses, in alphabetical order (7). They quickly noted that injection of Ad 12 into newborn hamsters resulted in tumors at the site of injection within a few months. This finding raised the interest in Ads tremendously, and it was quickly demonstrated that Ad 12 is able to transform baby hamster kidney cells *in vitro* (8).

### The early years

Studies of the Ad proteins were central in the 1960s. The capsid components were fractionated and purified in several laboratories using state-of-the-art biochemical techniques. This work formed the subject of my PhD thesis, which I defended in 1970 (9–12). From biochemical studies it became apparent that the virus must contain proteins other than hexons and pentons. Amino acid analysis revealed that the composition of the hexon, which should account for 95% of the protein mass of the virus, was similar to that of complete virions except for two amino acids, arginine and lysine. Studies by Laver et al. (13) and Prage et al. (14) showed that Ad DNA could be isolated as a DNA protein complex. Further studies revealed that the Ad chromatin contains two proteins: one smaller very basic component, and another moderately basic larger component. Pioneering studies on the peptide composition of the adenovirion were made by Jacob Maizel, who had introduced SDS polyacrylamide gel electrophoresis as a tool for the characterization of virion components. He identified 10 Ad polypeptides in his electropherograms, which with the current nomenclature are named polypeptides II, III, IIIa, IV, V, VI, VII, VIII, IX, and X (15). Polypeptides V and VII are highly basic and constitute the Ad chromatin together with the very small polypeptide X (also known as polypeptide Mµ). Polypeptide IX is associated with hexons in the facets of the virion, whereas polypeptides VI and VIII are located inside the capsid although their precise locations are as yet undetermined. Sixty copies of polypeptide IIIa are present in adenovirions, probably associated with the inside of the vertices. Anderson et al. showed that some of them are synthesized as larger precursors which during capsid assembly are cleaved by an Ad-encoded proteinase (16). Today it is known that polypeptides IIIa, VI, VII, VIII, and X are produced as precursors. Later work has shown that a few copies of additional proteins are present in the adenovirion, namely IVa2 and C-168 (17). Moreover, comprehensive studies of the spliced forms of Ad mRNA give room for additional viral proteins (18).

The 1970s was the decade of the Ad genome. Early work (19,20) had established that Ads consist of 13% double-stranded linear DNA and 87% protein with a molecular size ranging between 24 and 26 × 10^6^ (19,20). A noteworthy finding was that although the genomes of the human serotypes are very similar in size, they differed dramatically in GC contents, subgroup A being lowest with 47%–49% GC and subgroups C, D, and E the highest with 57%–59% GC (21).

Garon et al. (22) and Wolfson and Dressler (23) showed that single strands of Ad DNA form circles with so-called pan handles due to the presence of inverted terminal repetitions with a length of about 150 bp (24). Another unique property of the Ad genome is that the ends of the double-stranded DNA are covalently linked to a protein, the terminal protein, which plays a role in the initiation of DNA replication (25). Like some of the other capsid proteins, it is subjected to cleavage during virion maturation. Ad encodes its own DNA polymerase (26), and when the genome was sequenced it became apparent that it also encodes the terminal protein in a transcription unit which includes genes engaged in the replication of the Ad genome.

Replication of Ad DNA follows a unique pathway. As a postdoc I began to study replicating Ad DNA by CsCl gradient centrifugation and observed that replicating molecules had a greater density than mature viral DNA and that the difference was eliminated after treatment with a single-strand specific exonuclease (27). These observations fitted the model published by Sussenbach et al. proposing that Ad replication occurs by a strand displacement mechanism (28). Further studies showed that replication starts at either end of the genome and involves two steps (28). A viral-encoded DNA polymerase displaces first one of the strands, which is subsequently replicated in a second step. A puzzling observation is that bacteriophage phi29 employs an identical mechanism for its genome replication.

**Figure 2.**
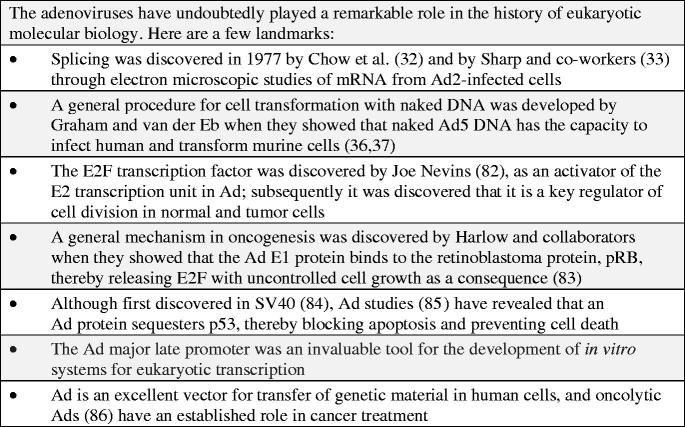
The iconic status of adenoviruses.

In the early 1970s little was known about viral genome organizations as no DNA mapping tools were available. Nothing was known about gene numbers and gene locations on the Ad chromosome. In the fall of 1971, I attended the DNA tumor virus workshop in Cold Spring Harbor. In one of the sessions Daniel Nathans gave a presentation during which he described how the SV40 genome could be cleaved into a distinct number of fragments by an endonuclease from *Hemophilus influenzae* (now known to be a mixture of Hind II and III) and that these could be separated by polyacrylamide gel electrophoresis. Nathans’s presentation was an eye opener. It clearly showed that the restriction enzymes would be ideal tools for mapping the chromosomes in DNA viruses. In the summer of 1971, I had arrived in Cold Spring Harbor as a postdoctoral fellow. One of my colleagues, Carel Mulder, was looking for an enzyme that would cleave circular SV40 DNA at one unique point. Herbert Boyer and his student Yoshimori had generously provided Mulder with a tube of an enzyme, now known as EcoR1, which had this specific feature. As I happened to have purified Ad2 DNA in store we decided to collaborate. The results we obtained were clear-cut; our friend Hajo Deluis showed with his electron microscope that the enzyme generated six fragments of different sizes (29), which due to differences in their denaturation profiles could be ordered along the linear genome. Richard Roberts arrived in Cold Spring Harbor the same year as I did. He set up a ‘factory’ for production of restriction enzymes with different cleavage specificities. Using some of these it became possible to cleave the Ad genome into sets of overlapping fragments which were ideal tools for gene mapping. From early studies it was known that a distinct set of Ad genes are expressed before replication of the genome and another after DNA replication had commenced, the so-called early and late genes. Transcriptional maps for Ad2 were constructed by Flint and co-workers (30) and by Clark Tibbetts and myself (31). The complementary strands of fragments generated by restriction enzymes EcoR1, Hind III, and Hpa1 were hybridized with mRNA isolated either early or late after infection. The results revealed an unexpected complexity with genes located on both complementary strands. The early genes were found in four locations, designated E1, E2, E3, and E4. Two were located on the so-called r-strand and two on the l-strand, whereas the bulk of the late mRNAs hybridized to the r-strand. Ad gene mapping was revolutionized when RNA-loop technology was introduced for mapping RNA transcripts. The application to Ads was pioneered by Chow and Broker, who constructed complete maps of Ad mRNAs with this technology (32). The technology paved the way for the discovery of RNA splicing in 1977 (33,34). This is one of many iconic discoveries in molecular biology which Ad researchers have made (Figure 2).

In the fall of 1976, while visiting the Cold Spring Harbor Laboratory, I attended an improvised seminar given by Walter Gilbert. He described a revolutionizing DNA-sequencing method based on chemical cleavage of end-labeled fragments prior to separation of the resulting fragments by polyacrylamide gel electrophoresis—later called the Maxam and Gilbert method. Although the method was unpublished until a year later (35), Gilbert handed out a detailed protocol. I received a copy, which I gave to one of my many talented PhD students, Göran Akusjärvi, when I returned to Sweden. Ten years later we published the complete 35,937 nucleotides long sequence of Ad2 together with Richard Roberts and his team (36).

Genes involved in Ad oncogenesis have been studied using different approaches. Pioneering work was performed by Graham and van der Eb. Their first aim was to develop a method for efficient transfer of Ad DNA to mammalian cells. After systematically testing a multitude of reagents, they discovered that Ad DNA is efficiently transferred to cells using calcium phosphate precipitation (37). This is a true landmark in the history of eukaryotic molecular biology as the method has proven to have a very broad scope. Using the method, they showed that naked Ad DNA can infect human cells and transform murine cells (38). They showed that the transforming genes are very sensitive to exonuclease treatment, indicating that they are located near one of the ends (39). Further on, they managed to transform baby rat kidney cells with a short DNA fragment, located at the left-hand end, only representing a few percent of the genome (40). As an extension of the work they succeeded in transforming human cells (41). This was unexpected, since human cells are permissive, allowing the virus to replicate, which leads to cell death. After many trials, they managed to establish the so-called 293 cell line which is transformed by Ad5 and only contains a subset of the viral genome, including the left-hand end. Through the history of molecular biology it has played an important role as a widely used research tool, among other things allowing mutants with defects in transforming genes to be constructed (42). Another approach to identify transforming genes involved studies of Ad-transformed cell lines. One method was to search for the presence of viral sequences in cell line 8617, a rat embryo cell line transformed by Ad2. DNA reassociation kinetics is a powerful method for detection of viral sequences in cells transformed by DNA viruses. The technology is simple and involves mixing radioactively labeled viral DNA with DNA from transformed cells. If viral sequences are present in the transformed cells, the speed of the reassociation of the labeled DNA will increase and will reflect the number of viral DNA copies in the transformed cell. In collaboration with Joe Sambrook, I applied the technology to 8617 cells. The results showed, contrary to what was reported, that only one copy of Ad2 DNA is present per cell (43). Further analysis showed that the resident genome is incomplete, lacking sequences from the middle of the genome (44). The study was extended by Gallimore et al. employing a bunch of independently Ad2-transformed cell lines (45). One of these was shown exclusively to contain sequences constituting 14% of the viral genome located at the left-hand end. Results from both approaches demonstrated that that the transforming genes of Ad are present in the E1 region of the genome. Subsequent studies revealed that region E1 comprises two transcription units, designated E1A and E1B.

The early Ad mRNAs have exceptionally complex structures and are partially overlapping, although they encode unique proteins. A variety of technologies have been used to study them, including Northern blotting, S1 nuclease analysis, electron microscopy, and cDNA cloning. Our contributions stem from a unique cDNA library which was constructed during a short visit to the Pasteur Institute in Paris.

In 1975 I had my first contacts with recombinant DNA. It started with a telephone call from Paris to Lennart Philipson. The reason for the call was that Luc Montaignier’s collaborator at the Pasteur Institute, Pierre Tiollais, had constructed a lambda bacteriophage (46), which could serve as a vector for propagation of foreign DNA. At the time, Boyer, Cohen, and Berg had caused a sensation by inserting DNA from the mammalian sources in an *E. coli* plasmid. Tiollais wanted to test if DNA from a human tumor virus would work. Recombinant DNA was an extremely controversial subject these days. The Asilomar conference took place in 1975, and Mayor Alfred Vellucci in Cambridge banned work with recombinant DNA in his town. From my visits to Cold Spring Harbor I could see the result in that Tom Maniatis came to the lab for experiments that were forbidden in his hometown. French law required a high-level safety laboratory for the experiment that Tiollais was planning. No such facility existed at the Pasteur Institute. Tiollais knew that Sweden at the time had a maximum-security laboratory at the so-called National Bacteriological Laboratory in Stockholm. Tiollais therefore came to Uppsala with his PhD student Michel Pericaudet. Phage constructs with the so-called B and F-fragments of Ad2 DNA were made in Uppsala and then transported to Stockholm. The experiment worked, and the recombinant phages replicated normally. The DNA was transcribed without generating any Ad protein. Today the experiment appears trivial. Then, however, recombinants between pro- and eukaryotes generated headlines. Pierre wrote a paper, which was submitted to *Cell*. Rumors tell that, due to the controversial nature of this kind of work, Ben Lewin sent the paper for review to more than 50 experts. Only one recommended publication, primarily because of ethical concerns. In retrospect it seems obvious that the paper lacked *Cell* qualities. In the end, Waclaw Szybalski, editor of *Gene*, allowed it to be published in *Gene* (47).

Some years later, the situation was reversed. In Uppsala we were interested in cloning the sites where the viral sequences are integrated in Ad-transformed cell lines. Now the recombinant DNA debate in Sweden had reached its peak, and we were unable to do the experiment at home. In the meantime a special self-contained laboratory, called the ‘submarine’, had been constructed at the Pasteur. I contacted my friend Tiollais who let us come to the Pasteur for our experiments. I spent five days in Paris mostly making libraries with DNA from transformed DNA inserted into the French lambda vector. Unfortunately, without success. However, something else turned out to be a success. With me to Paris I brought a tube with polyadenylated RNA from Ad-infected cells. Benefiting from the collegiality and expertise at the Pasteur, I for the first time in my life made cDNA constructs and cloned them in *E. coli*. On my final day in Paris, while the taxi was waiting to take me to the airport, I ran into Pericaudet who sported a big grin. He advised me to check the bacterial plates, which were loaded with clones. The library was then transferred to Sweden where we started to sequence clones from the transforming E1A region. The structures of the two longer E1A messages were unraveled quickly. A manuscript was prepared and submitted to *Nature*, which accepted the paper without revisions. The library turned out to be a goldmine for studies of the ‘zoo’ of early Ad mRNAs, and my PhD students did a great job with cDNAs from regions EIB, E3, and E4 (44,45,48–51).

## Adenovirus in the ‘omics’ era—a multi-pronged strategy

### A transcriptomic view of how host cells are reprogrammed

**Figure 3.**
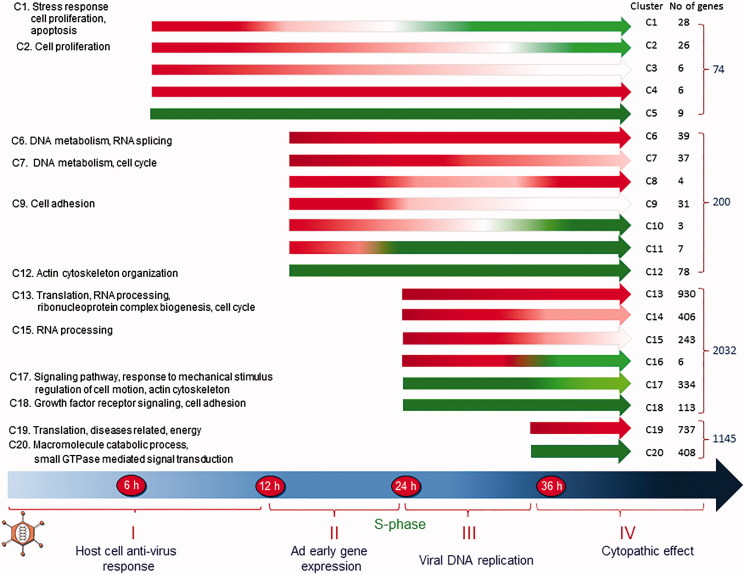
The figure illustrates the kinetics of transcription changes. The differentially expressed genes are grouped into 20 clusters (C1–C20). Red parts of the arrows indicate transcriptional up-regulation, and the green parts indicate suppression. Modified from (57).

At the turn of the century, after a two decades’ long detour into human genetics, I decided to re-enter the Ad field. The plan was to use the emerging ‘omics’ technologies to study how Ad-infected cells become reprogrammed during the course of a replication cycle. At the time, microarray analysis had emerged as a powerful technology to study gene expression in eukaryotic cells. No commercial microarray chips were, however, available when we started. Instead homemade chips needed to be produced, employing very expensive equipment for spotting DNA from clones containing DNA sequences with known origins. The method was cumbersome and imprecise, often requiring confirmation of the results by PCR. Luckily, a young, very skilled virologist, Hongxing Zhao, had just completed her postdoc and joined my group. She had the expertise and stamina to get the project rolling. First, we (52,53) and others (54) used microarrays, later switching to cDNA (55) sequencing to monitor cellular gene expression. To study changes occurring during the first 2 h of an infection Zhao et al. used microarray analysis (56). A dozen up-regulated genes with known functions were identified. Eight of these encode transcription factors. A set of five, ATF3, ATF4, KLF4, KLF6, and ELK 3, are linked to growth inhibition. Another set of two, NR4A1 and CEBPB, are involved in immune activation. The study was extended to other time points, switching to paired-end sequencing of cDNA copies of RNA, collected at four different times (57). The results generated a set of cellular gene expression profiles (Figure 3). Over all, about 7000 cellular genes are expressed at a measurable level at all time points, and a large fraction of the genes is changed more than or equal to 2-fold as compared to their expression in non-infected cells. Few changes in RNA expression occur early, as only 74 and 200 genes are differentially expressed at 6 and 12 hpi, respectively. Most changes take place at 24 hpi, when infection proceeds into the late phase. Then about 2200 additional genes become differentially expressed, most of which are up-regulated (Figure 3). At 36 hpi fewer additional genes are changed in their expression, and many of them are suppressed.

The differentially expressed genes fall into 20 major clusters based on the kinetics of their changes (Figure 3). Analysis using the Database for Annotation, Visualization, and Integrated Discovery software show that more than 90% of genes that are transiently up-regulated at 6 hpi are involved in the regulation of cellular proliferation, antiviral response, and cellular signaling. Genes in clusters 1 and 2 decay quickly and become suppressed later in infection. Genes encoding cytokines, active in immune response and cell growth control, form the most significant group. It is expected that genes showing similar expression profiles are regulated by common transcription factors (TF) or TFs from the same family. Thus, the genes in the 20 different clusters have been subjected to analysis for the presence of consensus TF binding sites in their promoter regions. For the genes in cluster 1, NFκB and c-Rel binding sites are prominent, as expected, as these are known to be present in genes engaged in immune response or apoptosis. They are hallmarks of most infections and play a critical role in virus-induced cytokine expression.

At 12 hpi viral gene expression has begun, redirecting cellular gene expression. Genes involved in DNA replication are now the most highly up-regulated group together with genes in the post-replicative DNA mismatch repair. Genes involved in transcription and pre-RNA processing are prominent in cluster 6, and genes implicated in cell cycle are significant in cluster 7, including key regulators for cell cycle progression from G1 to the S phase. Suppressed genes at this time are involved in antiviral response and in cell growth and proliferation. For the genes in clusters 6 and 7, the presence of the TF binding site for E2F stands out. This is expected as the Ad2 E1A protein binds pRb, thereby releasing E2F, which activates numerous genes essential for DNA replication and cell cycle progression. Expression of E2F itself is also up-regulated. Among eight E2Fs, five show increased expression at 12 hpi, reaching a maximum at 24 hpi before decreasing at 36 hpi.

Dramatic changes are seen at 24 hpi when infected cells are driven into the S phase. More than 2000 additional genes are now up- and down-regulated (Figure 3). Although most of them are similar in function to those up-regulated at 12 hpi, the number of genes involved in DNA metabolism and DNA replication increases nearly 10-fold. Genes in clusters 13, 14, and 15 include several DNA polymerases, replication factors, histones, and many cell cycle regulators. In addition, genes participating in RNA processing become up-regulated, like genes required for efficient export of polyadenylated RNA and genes encoding components of the exosome complex involved in the degradation and processing of a wide variety of RNA species. Based on their expression level at 36 hpi, the up-regulated genes at 24 hpi fall into four clusters, 13, 14, 15, and 16. Although many functions are shared between them, some differences exist. For the genes in cluster 13, the most significant function is protein translation, RNA processing, and cell cycle, whereas the most significant functions of the others are DNA metabolism and replication, cell cycle, and stress response. The E2F binding sites are common in clusters 13 and 14, as are the binding sites for GABP, NRF1, and ATF/CREB. GABP regulates genes that are involved in cell cycle control, protein synthesis, and cellular metabolism. NRF1 activates the expression of some key metabolic genes regulating cellular growth. The most significant TF binding sites for genes in cluster 15 differed in that the ATF/CREB binding motif becomes more abundant. The ATF/CREB family has diverse functions, controlling cell proliferation and apoptosis. The number of suppressed genes at 24 hpi also increases. They are involved in either cell motion and structure or growth mediated by growth factors. Gene functions involved in cytoskeleton organization are significant in cluster 17, whereas genes implicated in cell adhesion are present in cluster 18.

Fewer changes in cellular gene expression take place at 36 hpi when the infection has proceeded beyond the late phase and suppression is more pronounced (Figure 3). The most significant function of the up-regulated genes is protein translation, including genes for ribosomal proteins, translation initiation factors, and translation elongation factors. Genes involved in the generation of metabolites and energy, as well as oxidation/reduction, also become evident. The most outstanding function of the suppressed genes is the cellular macromolecule catabolic process including numerous genes mediating ubiquitination and subsequent proteasomal degradation. Another important function is signal transduction, involved in vesicle transport. At 36 hpi, different sets of TF binding sites are significant in the up-regulated genes, including SP1, STRA13, and NF-Y, in addition to GABP, while the binding sites for E2F become less important.

### A role for non-coding RNAs?

It was discovered more than 50 years ago that Ad encodes a small abundantly expressed RNA first named 5.5 SRNA, later VA RNA (58). Further studies revealed that VA RNA in Ad2 consists of two species, VA RNAI and VA RNAII (52,53). Their positions in the genome were mapped, and sequencing revealed that they are about 160 nucleotides long (59). Studies by Mathews, Shenk, and their co-workers revealed that the more abundantly expressed VA RNAI plays a critical role in Ad replication as it binds to and blocks the double-stranded RNA-activated protein kinase. The function of VA RNAII, which is absent in some Ads, is unknown. VA RNAI is cleaved by dicer, and the resulting MIVA RNAs are incorporated into RISC complexes (60). As many viruses encode their own microRNAs (miRNAs), we initiated an unbiased search for Ad2-encoded miRNAs, using deep cDNA sequencing of small RNAs expressed at different times after infection (56). The results revealed a set of small RNAs, constituting more than 1% of the total pool of small RNAs. They are between 25 and 35 nucleotides long and map in the region of the VA RNAI and RNAII genes, although not completely overlapping. A surprising observation is that some of the small RNAs are expressed very early before any other viral RNAs are expressed. Target predictions indicate that the small RNAs are involved in signaling pathways.

Changes in cellular microRNA (miRNA) expression during the course of an Ad2 infection in human lung fibroblasts have also been studied by deep RNA sequencing (61). More than 100 miRNAs with an altered expression were identified. The changes range from up-regulation of the miRNAs known as tumor suppressors (such as miR-22, miR-320, let-7, miR-181b, and miR-155) to down-regulation of oncogenic miRNAs (such as miR-21 and miR-31) early. Late the pattern is the opposite, with down-regulation of tumor suppressor miRNAs (such as let-7 family, miR-30 family, 23/27 cluster) and up-regulation of oncogenic miRNAs (such as miR-125, miR-27, and miR-191). The switch in expression pattern occurs when Ad DNA replication starts. It thus appears that miRNAs are engaged in growth inhibition early and stimulation of growth late.

The deregulation of cellular long non-coding RNA (lncRNA) expression during a human Ad infection has also been studied by deep RNA sequencing (62). Expression increased substantially following the progression of the infection. Thus, the expression of more than 50% of some 600 significantly expressed lncRNAs is changed more than 2-fold. Most of them are up-regulated and activated during the late phase. Based on the genomic locations of deregulated lncRNAs in relation to known mRNAs and miRNAs, they are predicted to be involved in many biological processes in the late phase, like growth, structure, and apoptosis. In the early phase wound healing is involved, while cell proliferation, protein synthesis, modification, and transport are scored in the intermediate phase. The most significant functions of cellular RNA-binding proteins known to interact with the deregulated lncRNAs are splicing, nuclear export, and translation. Based on the predictions it seems likely that Ads exploit the lncRNA network to optimize their reproduction.

Among eight E2Fs, five show increased expression at 12 hpi, reaching their maximum at 24 hpi before decreasing at 36 hpi.

### Mass spectrometry presents another view

My scientific career began in protein chemistry. I still have vivid memories of hours spent packing Sephadex, Agarose, and DEAE chromatography columns. Sequencing, using my countryman Edman’s method, was out of the question with the resources we had. Mass spectrometry for analysis of small molecules was available but not for molecules as large as polypeptides. In 2002, Fenn, Tanaka, and Wutrich received the Nobel Prize in chemistry for ‘the development of methods for identification and structure analysis of macromolecules’. From their Nobel lectures I became aware of the outstanding potential mass spectrometry offers for studies of proteins. It tempted me to return to my studies of the Ad proteins. Luckily, I managed to recruit a very skilled postdoc, Sara Lind (née Bergström), who mastered the technology. As a first step, we subjected highly purified Ad2 virions to mass spectrometry in a search for hidden proteins and posttranslational modifications (63,64). A number of modifications were detected. The pIIIa protein is the most highly modified protein with 18 verified phosphorylation sites, three nitrated tyrosine sites, and one sulfated tyrosine. Nitrated tyrosines and lysine acetylations are present in hexon and pVI as well. A study of non-structural Ad proteins unraveled the primary structures of the proteins predicted to be encoded by the viral genome. Twenty-seven non-redundant sites of phosphorylation on seven different non-structural proteins were identified. The most heavily phosphorylated non-structural protein is the DNA-binding protein with 15 different sites (65,66).

Our next step was to use mass spectrometry to monitor changes in cellular protein expression during the course of an Ad infection (67,68). State-of-the-art technology, i.e. stable isotope labeling of amino acids in cell culture (SILAC) combined with nanoLC-MS/MS, was used. About 2500 proteins were expressed at a measurable level, and approximately 25% and 35% of these proteins are altered early and late after infection. The proteins fall into different clusters based on their changes in abundance. At 6 hpi, proteins involved in cell-to-cell adhesion, cytoskeletal organization, protein folding, and prevention of protein aggregation or in the nucleolar-cytoplasmic transport stand out. One especially interesting protein, BNIP2, interacts with the Ad-encoded antiapoptotic 19 kDa protein.

Most proteins, uniquely altered at 12 hpi, are up-regulated, and several different pathways are overrepresented by them. Remodeling the cytoskeleton stands out, and many up-regulated proteins are involved in cytoskeletal regulation by Rho GTPase as well as inflammation mediated by chemokine and cytokine signaling, and integrin signaling. The toll receptor signaling pathway is deregulated at this time, although the activation increases at 24 hpi. Toll-like receptors play critical roles in the innate immune system by recognizing pathogen-associated molecular patterns derived from various microbes (69,70). Other altered proteins are involved in the regulation of the glutathione metabolism and detoxification processes, in the organization of the proteasome system, and in signaling pathways such as TGF-beta, Rho, and NFκB. The stress response proteins HSPA1A/B are mildly altered at 12 hpi before becoming the most highly up-regulated protein at 24 hpi. Another deregulated pathway is fructose galactose metabolism. This makes sense as proteins in this pathway are involved in the production of precursors to be used in the glycolysis cycle for energy production.

The most highly suppressed proteins at 12 hpi are collagens and proteins related to them. In most cases, the suppression was sustained over time. Another suppressed protein at 12 hpi is SERPINE1, the principal inhibitor of tissue plasminogen and urokinase activators. Another interesting pathway overrepresented at 12 hpi is the PDGF pathway. The suppressed plasminogen and the urokinase activators are needed for activation of the pathway, indicating that the cell tries to block activation of the PDGF pathway. The PDGF receptor alpha is suppressed at all time points of the infection, the beta receptor only at late time points. These receptors also bind adaptor molecules which form complexes with other signaling molecules, such as the regulatory subunit p85 of the phosphatidylinositol 3′-kinase, and GRB2, which binds the nucleotide exchange molecule SOS1, thereby activating RAS and the ERK MAP-kinase pathway. These events apparently take place early in infection before the PDGF pathway is shut down. The activation of PI3K, a consequence of the early virus–cell interactions, leads to actin reorganization via the GTPase activator for the Rho, Rac, and Cdc42 proteins. These proteins are up-regulated in the early phase.

At 24 hpi the pentose phosphate pathway is up-regulated at the protein level but not at the mRNA level (71). This pathway cooperates with glycolysis for energy production. At late times the virus needs high amounts of nucleotides for its replication, and therefore it requires ribose-5-phosphate. Serine synthesized from the glycolytic intermediate 3-phosphoglycerate is converted to glycine, which is an important precursor for purine biosynthesis, via the serine glycine biosynthesis pathway (72). Because of its conversion to glycine, serine is also the donor of folate-linked one-carbon units which are required for nucleotide biosynthesis (73). At 6 hpi the serine glycine biosynthesis pathway is activated while the *de novo* purine biosynthesis and *de novo* pyrimidine ribonucleotides biosynthesis pathways are activated at all time points, most highly late, like the glycolysis pathway. The FGF and the EGF signaling pathways are activated at 6, 12, and 24 hpi but become down-regulated later. The inactivation of these signaling pathways might be caused by the down-regulation of their ligands at the mRNA level 24 hpi (55). The expression of several proteins involved in the integrin signaling pathway, including several integrins, collagens, and RAS family members, increases first, but most genes encoding these proteins are down-regulated at 24 and 36 hpi at the mRNA level (71).

A number of transcription factor (TF) binding sites have been identified in genes deregulated at the protein level at different time points. MYC is highly activated, and its activation increases with time. This explains why the glycolysis and the pentose phosphate pathways are overrepresented in spite of the decreased level of MYC mRNA. Another transcription factor, activated at intermediate and late time points, is E2F. Several components in the DNA and cell cycle machinery are up-regulated in parallel with their mRNAs at 24 hpi, but not at 12 hpi. E2F-activated transcription begins at 12 hpi, while changes at the protein level are noted first at 24 hpi.

Transcriptomic data reveal an up-regulation of genes controlled by the ATF/CREB family at 24 hpi (55). These transcription factors control the expression of genes involved in DNA and RNA metabolism, but also genes involved in the stress response. TF analysis of the proteomic data confirmed the activation of CREB1 at late time points. Cellular defense against ROS is controlled by NRF2, highly activated at all time points. It inhibits lipogenesis, activates the oxidation of fatty acids, simplifies the flux through the pentose phosphate pathway, and increases NADPH regeneration and purine biosynthesis.

### Divergent mRNA and protein expression

The correlation between changes in RNA and protein expression in Ad2-infected cells is surprisingly low (57). Sets of discordantly expressed genes have been analyzed with focus on genes involved in important pathways, like immune response and cell cycle and growth control. NFκB and c-Rel binding sites are frequent in promoter regions of the genes that are transiently up-regulated during the early phase, showing that NFκB signaling plays a crucial role in the cellular defense. Transcription of most genes involved in this pathway is induced during the early phase, but becomes down-regulated later. RELA and IkBKB, an inhibitor of NFκB kinase, have been studied at the protein level. They show, unexpectedly, opposite expression profiles compared to their mRNAs. The IkB kinase interacting protein IkBIP also shows divergent RNA and protein expression profiles. Thus, it seems that the NFκB pathway remains activated at the protein level, while transcription is down-regulated. Target genes, containing NFκB and cRel binding sites in their promoter regions, are, however, suppressed when infection progresses, indicating that the NFκB proteins are functionally inactivated by yet unidentified posttranslational mechanisms. Thus, there must be additional regulatory steps involved in the NFκB pathway.

JAK/STAT signaling plays an important role in cellular immune response and growth control. Many genes involved in this pathway are deregulated (55,74), including JAKs, STATs, and their negative regulators, as well as the importin α-5 and the Ran nuclear import pathway. Most of them are down-regulated transcriptionally. A few proteins in this pathway are detectable at the protein level, and the results are surprising. In contrast to the transcriptional down-regulation of STATs, all detected STAT proteins—including STAT1, 3, and 6—are up-regulated at 24 and 36 hpi. The JAK and STAT negative regulators are also detectable. Expression of the PTPN11 protein is increased at 24 and 36 hpi, although its RNA remains unchanged during the infection. At 24 hpi the PTPN1 protein decreases slightly, while its RNA level is reduced nearly 4-fold. Surprisingly, most STAT proteins seem to be inactivated, since no significant up-regulation of genes containing a STAT consensus sequence is detected. Furthermore, expression of IFNα, a well-known target gene in the STAT pathway, decreases during the infection. Several explanations are conceivable. Like most other activators, the functions of STAT proteins depend on other site-specific DNA-binding proteins, such as p300/CBP, which is sequestered by the E1A protein. In addition, posttranslational modifications, including tyrosine phosphorylation, have an impact on STAT function. Furthermore, nuclear transport of STAT is strictly regulated. This explanation is unlikely, however, since importin α-5 and Ran, required for the entry of STAT to the nucleus, are up-regulated at both the RNA and protein levels at 24 and 36 hpi.

Apoptosis pathways are extensively deregulated during Ad2 infection. The early protein E1A induces p53-mediated apoptosis. This is subsequently counteracted by the Ad-encoded E1B proteins, E1B-55K and E1B-19K. By directly binding to p53, E1B-55K suppresses p53-dependent transcription and promotes p53 degradation through an E4orf6-E3 ubiquitin ligase complex (75). The E1B-19K, which is a homolog of BCL-2, binds to BAK and abrogates the interaction of BAK with BAX. It prevents the conformational changes that activate BAX and the release of cytochrome c (67). Moreover, Ad2 E3 gene products inhibit apoptosis induced by death receptor signaling (68). The caspases and the Bcl2 family are key players in apoptosis. Deregulation of caspase 3 and several genes belonging to the Bcl2 family takes place at both the RNA and protein levels. At the transcriptional level, six out of eight caspases are down-regulated. Only Casp3 is detectable at the protein level with an expression profile opposite to that of its RNA at 24 and 36 hpi. At 36 hpi, the Casp3 RNA level had decreased more than 3-fold, while its protein level is increased 1.7-fold. Thus, several important players in apoptosis are up-regulated, while their functions must be blocked since apoptosis is efficiently inhibited during an Ad2 infection. In fact, several well characterized Casp3 targets were up-regulated at the protein level. Ad has obviously established efficient ways to counteract the apoptotic pathways by encoding their own antiapoptotic genes, thereby preventing cell death before the replication cycle is completed.

### Epilogue

Historically, the Ad replication cycle is divided into two phases separated by the onset of viral DNA replication. Based on temporal changes in cellular RNA expression patterns, an Ad2 infection in primary lung fibroblasts can be divided into four periods. The changes follow a logical pattern. Already at 1 hpi a set of transcription factors are up-regulated, primarily involved in growth inhibition and cellular defense. A few hours later genes involved in cytokine expression and apoptosis are prominent. At 12 hpi genes involved in the cell cycle, DNA synthesis, and replication and RNA splicing dominate, while genes engaged in the actin cytoskeleton are suppressed. At 24 hpi the repertoire is enlarged with genes involved in RNA processing and translation, while genes involved in growth factor receptors, cytoskeleton organization, and cell adhesion are suppressed. At 36 hpi the repertoire is complemented with genes engaged in energy production and oxidation/reduction, whereas GTPase-mediated signal transduction is suppressed.

Conclusions drawn from the transcriptomic data are supported and complemented by the proteomic data. Differences do, however, exist, as several important deregulated pathways fail to show up in one of the analyses. Serine glycine biosynthesis and mannose metabolism are two examples. Overall, pathways related to energy and biosynthesis of nucleic acid precursors failed to stand out in the transcriptomic analysis. The PDGF, EGF, and FGF pathways are suppressed at both levels, although the proteins decay more slowly than their mRNAs. An interesting pathway, activated already at 6 hpi, is the cytoskeletal regulation by Rho GTPase. This pathway has been studied during Ad infection by other methods (76). After the attachment to its receptor CAR and the αvB5 integrin, virus endocytosis requires reorganization of the host cell actin cytoskeleton. These pathways are interlinked with the toll-like receptor pathway (77,78), which does not show up in the transcriptomic analysis.

The proteomic (unlike the transcriptomic) analysis identified an activated MYC pathway. Stabilization of MYC requires the p400 protein, and E1A promotes the association of MYC and p400 at MYC target genes (79). Different studies have characterized virus-induced changes in host cell metabolism (80), and it has been suggested that Ad E4orf1 binds to MYC and induces the expression of glycolytic target genes and nucleotide biosynthesis from glucose intermediates (81). Therefore, the activation of MYC by E1A and E4orf1 explains why the glycolysis and the pentose phosphate pathways are overrepresented in spite of the decreased level of MYC mRNA.

A most puzzling observation in our studies concerns the immune defense pathways. Some of them are up-regulated early at both the RNA and protein levels. RNA expression decays at later time points, as would be expected. Surprisingly the protein levels are sustained or enhanced late after infection. Still the target genes remain suppressed, showing that the proteins are inactivated by hitherto unknown mechanisms.

Overwhelming evidence shows that non-coding RNAs of different kinds play important regulatory roles in mammalian cells. The miRNAs act at the posttranscriptional level and regulate translational efficiency. The predicted functions of the miRNAs that are deregulated early and late after infection imply that there is a cooperation between the miRNAs and the gene products that have been identified in the transcriptomic and proteomic analyses. Early miRNAs would inhibit cell growth by suppression of oncogenic miRNAs and up-regulation of miRNAs acting as tumor suppressors. Later the situation is reversed, i.e. oncogenic miRNAs are up-regulated while tumor-suppressing miRNAs are down-regulated.

As many as 600 lncRNAs seem to be deregulated during an Ad2 infection. Their predicted functions imply that they could be involved in many steps of the replication cycle, although experimental proof remains to be provided.

Taken together, available results demonstrate that Ad replication involves many more regulatory steps than believed. A conclusion is also that transcriptomic and proteomic studies complement each other and allow a more complete picture of a viral replication cycle to emerge.
